# CDK9 and its repressor LARP7 modulate cardiomyocyte proliferation and response to injury in the zebrafish heart

**DOI:** 10.1242/jcs.175018

**Published:** 2015-12-15

**Authors:** Gianfranco Matrone, Kathryn S. Wilson, Sana Maqsood, John J. Mullins, Carl S. Tucker, Martin A. Denvir

**Affiliations:** 1 British Heart Foundation Centre for Cardiovascular Science, The Queen's Medical Research Institute, The University of Edinburgh, Edinburgh EH16 4TJ, UK; 2 Center for Cardiovascular Regeneration, Department of Cardiovascular Sciences, Methodist Hospital Research Institute, Houston, TX 77030, USA

**Keywords:** Zebrafish, CDK9, LARP7, Heart, Repair

## Abstract

Cyclin dependent kinase (Cdk)9 acts through the positive transcription elongation factor-b (P-TEFb) complex to activate and expand transcription through RNA polymerase II. It has also been shown to regulate cardiomyocyte hypertrophy, with recent evidence linking it to cardiomyocyte proliferation. We hypothesised that modification of CDK9 activity could both impair and enhance the cardiac response to injury by modifying cardiomyocyte proliferation. Cdk9 expression and activity were inhibited in the zebrafish (*Danio rerio*) embryo. We show that dephosphorylation of residue Ser2 on the C-terminal domain of RNA polymerase II is associated with impaired cardiac structure and function, and cardiomyocyte proliferation and also results in impaired functional recovery following cardiac laser injury. In contrast, de-repression of Cdk9 activity, through knockdown of La-related protein (Larp7) increases phosphorylation of Ser2 in RNA polymerase II and increases cardiomyocyte proliferation. Larp7 knockdown rescued the structural and functional phenotype associated with knockdown of Cdk9. The balance of Cdk9 and Larp7 plays a key role in cardiomyocyte proliferation and response to injury. Larp7 represents a potentially novel therapeutic target to promote cardiomyocyte proliferation and recovery from injury.

## Abstract (precis)

**Summary:** The balance of CDK9 and LARP7 plays a key role in cardiomyocyte proliferation and response to injury. LARP7 represents a potentially novel therapeutic target in promoting recovery from injury.

## INTRODUCTION

Cyclin dependent kinase 9 (CDK9) has emerged from the wider family of CDKs as an important signaling mechanism in the initiation and progression of cardiac hypertrophy (Krystof et al., 2012, 2010). Sano and colleagues (2002) have shown that CDK9 expression is upregulated and activity is enhanced during cardiac hypertrophy, and that chronic activation of CDK9 predisposes to heart failure in adult mouse myocardium. More recently, CDK9 has been shown to act as a binding partner of GATA4, an important regulator of cardiomyocyte proliferation in mammals and zebrafish (Kikuchi et al., 2010). This link to GATA4 provides support for a possible role of CDK9 in regulating cardiomyocyte proliferation (Sunagawa et al., 2010).

In mammalian species, cardiomyocytes proliferate rapidly during fetal life but lose this capacity within the weeks and months following birth, leaving only a very low level of background mitosis in adult hearts (Ahuja et al., 2007). The zebrafish heart, by contrast, retains a much higher rate of background cardiomyocyte cell division throughout adulthood, which is associated with a remarkable capacity to regenerate cardiac tissue following injury (Poss et al., 2002). An additional contrasting feature of the zebrafish heart is that, unlike the mammalian heart, it appears to lack a classic hypertrophic response, although few hypertrophic models have been developed in the fish (Sun et al., 2009). Indeed, Becker and colleagues (2011) have shown that a Troponin T (*tnnt2*) gene knockdown targeting the exon 13 splice donor site in embryonic zebrafish, designed to mimic human hypertrophic cardiomyopathy, results in cardiomyocyte hyperplasia and a dilated ventricle, despite a switch in the use of molecular pathways associated with hypertrophy.

Cardiac hypertrophy is typically associated with a global increase in mRNA and protein synthesis in cardiomyocytes. CDKs are a family of cell cycle regulators that are closely linked to transcription (Garriga and Grana, 2004; Loyer et al., 2005). Indeed, persistent endogenous CDK inhibition in adult cardiomyocytes has been proposed as one of the reasons why these cells rarely re-enter the cell cycle following injury or in response to haemodynamic, or other, stress. In support of this, knockdown of endogenous CDK inhibitors by using small interfering RNA induces neonatal and adult cardiomyocytes to re-enter the cell cycle, resulting in active proliferation of previously quiescent cells (Di Stefano et al., 2011). This intriguing finding suggests that there is a key role for CDKs in controlling the proliferative behaviour of cardiomyocytes and points to a potential mechanism by which cardiomyocytes could be induced to divide.

CDK9 becomes active by complexing with cyclin T, forming the positive transcription elongation factor b (P-TEFb), leading to enhanced transcriptional activity through RNA polymerase II. Inactivation results from binding to hexamethylene-bisacetamide-induced protein (HEXIM) 1 and/or HEXIM2 (Barboric et al., 2005; Yik et al., 2003) and the La-related protein 7 (LARP7) (He et al., 2008; Markert et al., 2008). LARP7, also called PIP7S (He et al., 2008), is a highly specialised RNA-binding molecule that stabilises the CDK9 inactivating complex by providing a bridge between 7SK-RNA and HEXIM proteins. Indeed, a previous study (Markert et al., 2008) using silencing-RNA against LARP7 caused upregulation of RNA polymerase II activity. LARP7 therefore appears to be an important regulator of CDK9 and consequently of P-TEFb complex activity (Krueger et al., 2008).

These regulatory mechanisms provide novel and important insights into ways in which CDK9 could be manipulated for therapeutic benefit. Indeed, although pharmacological CDK9 inhibition has been proposed as a therapy for abrogating cardiac hypertrophy (Krystof et al., 2010), the possibility of enhancing CDK9 activity has been overlooked as a mechanism that could be used to induce cardiac hypertrophy and to stimulate cardiomyocyte proliferation. CDK9 is therefore a potentially important pathway that merits further evaluation as a therapeutic target.

In this study, we modulated Cdk9, both genetically and pharmacologically, in the zebrafish embryo. We show that genetic knockdown and pharmacological inhibition of Cdk9 inhibits cardiomyocyte proliferation, and reduces cardiac function and the ability of the heart to recover following cardiac laser injury. Conversely, genetic inhibition of Larp7, a Cdk9 repressor, provoked both a hyperplastic and hypertrophic cardiac response with preservation of cardiac function following injury, and rescued the effects of CDK9 knockdown.

## RESULTS

### Cdk9 and Larp7 expression display distinct patterns in whole larvae and in isolated larval hearts during normal development

**Fig. 1.**
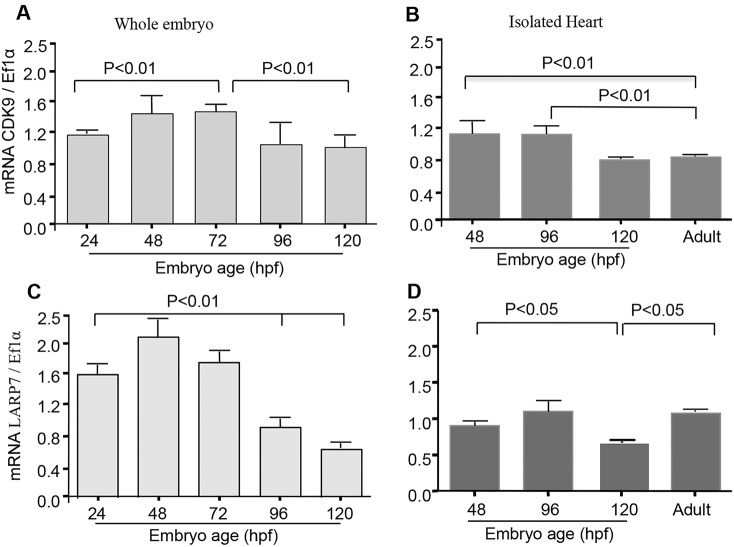
**qPCR assessment of *cdk9* and *larp7* gene expression in the whole embryo and in isolated zebrafish hearts.** *cdk9* and *larp7* mRNA relative abundance in whole embryos collected between 24 and 120 hpf (A,C), and in isolated embryonic hearts at 48, 96, 120 hpf, as well as in adult heart (6 months of age) (B,D). Data were compared using one-way ANOVA followed by Bonferroni's multiple comparison test. Each time-course was studied in one clutch of eggs with at least 10 embryos per time point. Mean values represent three clutches of eggs. Error bars represent s.e.m.

Cdk9 was detected in normal whole larvae from 24 h post fertilisation (hpf) and showed a distinctive pattern of expression during the course of development, increasing between 24 and 72 hpf (*P*<0.01) and then decreasing towards 120 hpf (Fig. 1A). In pooled isolated embryonic hearts (*n*=150 per sample, *n*=3 samples), Cdk9 mRNA peaked at 48 hpf and again at 96 hpf, decreasing at 120 hpf to levels then detected in adult isolated hearts. Cdk9 was also detectable at moderate levels (relative to those in the embryo) in the adult heart (Fig. 1B). In whole larvae, Larp7 expression reached a peak at 48 hpf and then decreased at 72, 96 and 120 hpf (Fig. 1C). In isolated embryonic hearts, Larp7 mRNA levels increased slightly between 48 and 96 hpf, and then reduced at 120 hpf. In the adult heart, Larp7 mRNA was also detectable (Fig. 1D).

### Survival and gross phenotype of flavopiridol- and Cdk9-morpholino-treated larvae

Incubation of embryos in flavopiridol at less than 24 hpf resulted in high levels of mortality, and so we used continuous incubation from 24 hpf, which resulted in a modest reduction in survival in treated embryos compared to that of controls at 120 hpf (77±3% vs 91±4%, respectively, *P*>0.05; Fig. S1A).

Absence of heart beat and lack of tail blood flow combined with other major structural malformations were the criteria used to evaluate mortality. These larvae also displayed an excess of body axis deformities, a small head, pericardial oedema and reduced tail blood flow. Flavopiridol had no significant effects on the anatomical configuration of the heart.

Treatment with a morpholino that blocked the ATG translation of *cdk9* (Cdk9-Mo-TB) resulted in a significant reduction in survival compared to that of controls at 120 hpf (47±4.5% vs 85±4%, respectively, *P*<0.001; Fig. S1B). In contrast, treatment with a morpholino that blocked the splicing of *cdk9* (Cdk9-Mo-SB) was associated with a higher level of survival compared to that of controls (72±5% vs 85±4%, respectively, *P*>0.05). Larvae that had been treated with the morpholinos against *cdk9* displayed an excess of developmental and structural abnormalities, compared to controls, including deformity of trunk and tail curvature, a small head, pericardial oedema, reduced tail blood flow and global developmental delay compared with controls (Fig. S2). These abnormalities were less frequent upon treatment with Cdk9-Mo-SB compared to treatment with Cdk9-Mo-TB. This is a fairly typical observation because translation-blocking morpholinos are known to inhibit both maternal and zygotic transcripts (Nasevicius and Ekker, 2000), whereas splicing-blocking morpholinos act only on zygotic transcripts. Owing to the high mortality associated with the Cdk9-Mo-TB, we preferentially used embryos that had been treated with Cdk9-Mo-SB, which showed a similar degree of Cdk9 protein knockdown, during the majority of the laser injury and rescue experiments to avoid the confounding issue of selection-survival bias.

### Effects of Cdk9 inhibition on *cdk9* gene transcription, protein expression and phosphorylation of Ser2 on RNA polymerase II

**Fig. 2.**
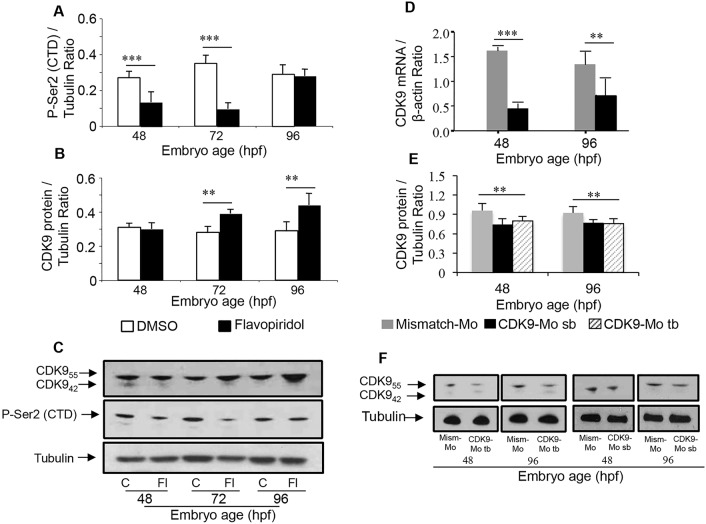
**Effect of Cdk9 inhibition on Cdk9 levels and phosphorylation of RNA polymerase II at Ser2.** Effects of flavopiridol (A–C) on the downstream phosphorylation of Ser2 in the C-terminal domain of RNA polymerase II [P-Ser2 (CTD)] (A) and on Cdk9 protein expression at 48, 72 and 96 hpf (B). Lower panels show sample western blot membranes after Flavopiridol (C) and Cdk9-Mo (F) treatment showing Cdk9 isoform 55 kDa (CDK9_55_), which is dominant over the expression of the smaller isoform at 42 kDa (CDK9_42_) Cdk9 mRNA (D) and protein (E) levels were significantly reduced at 48 and 96 hpf following injection of Cdk9-Mo-SB or Cdk0-Mo-TB. β-actin mRNA was used to normalise mRNA levels, whereas tubulin was used to normalise protein level. (*n*=3 experiments, >10 embryos per group, **P<0.01, ***P<0.001, one-way ANOVA test followed by Bonferroni's post-hoc test). C, control; Fl, flavopiridol; Mism-Mo, mismatch morpholino; Cdk9-Mo-SB, CDK9 morpholino splice blocking; Cdk9-Mo-TB, Cdk9 morpholino translation blocking. Means±s.e.m. are shown in A, B, D and E.

Treatment with flavopiridol caused a reduction in the levels of the phosphorylated form of the serine 2 residue of the C-terminal domain in whole larvae compared to that in controls (Fig. 2A–C), as predicted because of its known pharmacological action (Chao et al., 2000; Bosken et al., 2014). However, flavopiridol-treated embryos, unexpectedly, showed an increase in Cdk9 protein in whole larvae at 72 and 96 hpf (Fig. 2B,C).

Larvae that had been injected with Cdk9-Mo-SB showed a marked reduction in the abundance of *cdk9* mRNA by over 70%, as expected with morpholino treatment (Fig. 2D). This resulted in about a 20% reduction in the levels of Cdk9 protein at 48 and 96 hpf (Fig. 2E,F). This reduction in Cdk9 protein was confirmed with both splice- and translation-blocking morpholinos (Fig. 2F).

### Cdk9 inhibition reduces size and function of the ventricle

**Fig. 3.**
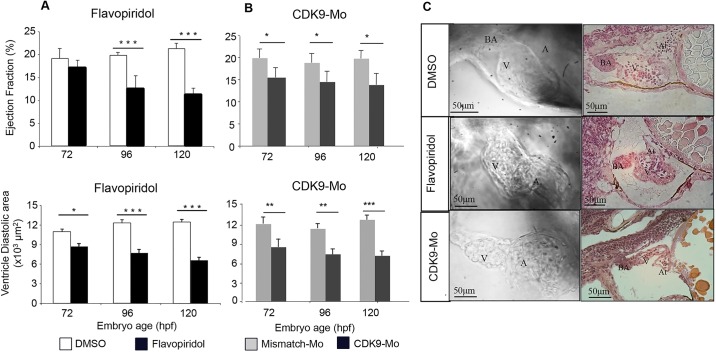
**Effects of CDK9 inhibition or knockdown on the structure and function of the developing zebrafish heart.** For pharmacological inhibition (A), embryos were continuously exposed to vehicle (1% DMSO, clear bars) or flavopiridol (3 μmol/l, dark bars) from 24 to 120 hpf. For morpholino-mediated knockdown (B), embryo eggs at the one- to two-cell stage were injected with Cdk9 mismatch morpholino (clear bars) or Cdk9-targeting morpholino (SB, black bars). Ventricle ejection fraction (A,B, upper panels) and diastolic area (A,B, lower panels) were assessed sequentially in the same embryos at 72, 96 and 120 hpf. Images in C show embryonic hearts following treatment with flavopiridol and Cdk9-Mo (SB). Left-hand panels are bright field images of the heart, and right-hand panels are histological sections through the heart stained with haematoxylin and eosin (H&E). *n*=3 experiments, >10 embryos per experiment, *P<0.05, **P<0.01, ***P<0.001. Two-way ANOVA test for repeated measures followed by Bonferroni's post-hoc test. ‘A’, atrium; BA, bulbous arteriosus; V, ventricle. Means±s.e.m. are shown in upper and lower panels of A and B.

During normal development, zebrafish larvae show a progressive increase in ventricle size, measured as diastolic area, in the period 72–120 hpf. The ejection fraction of the ventricle does not change during normal development. However, the increase in heart rate and cardiac size results in an increase in cardiac output. Pharmacological inhibition of Cdk9 with flavopiridol from 24 hpf onwards reduced ventricle size and the ejection fraction compared to controls (Fig. 3A), although flavopiridol did not have a great impact on the anatomical structure of the heart (Fig. 3C). Treatment with Cdk9-Mo-SB had a marked effect on cardiac structure during development with a high proportion of hearts showing a string-like appearance at 120 hpf (Fig. 3B,C). These hearts were also significantly smaller and displayed a reduced ejection fraction (Fig. 3B).

### Larp7 knockdown causes a mild cardiac phenotype with enhanced phosphorylation of Ser2 on RNA polymerase II

**Fig. 4.**
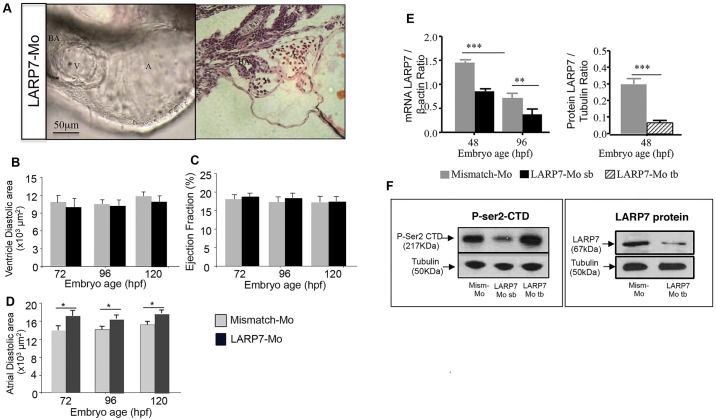
**Effects of Larp7 knockdown on cardiac structure and function.** (A) Larp7-targeting morpholinos [either splice blocking (SB) or translation blocking (TB)] resulted in normal heart development, here shown *in vivo* (left hand panel) and following H&E staining (right hand panel). Ventricular diastolic area (B) and ejection fraction (C) in Larp7-knockdown embryos were similar to controls (Larp7 mismatch morpholino). A mild dilatation of the atrium (D) was observed in Larp7-knockdown (TB and SB Larp7-Mo) embryos. Larp7 mRNA (E) and protein (F) were significantly reduced at 48 and 96 hpf. Larp7-Mo-TB (larp7 Mo) injection caused increased phosphorylation of Ser2 in the RNA polymerase II C-terminal domain (P-Ser2 CTD), suggesting an upregulation of Cdk9 activity. *n*=3 experiments, *P<0.05, **P<0.01, ***P<0.001, two-way ANOVA followed by Bonferroni's post-hoc test. Means±s.e.m. are shown in B, C, D and E.

Larvae that had been injected with a morpholino blocking Larp7 splicing or translation (Larp7-Mo-SB and Larp7-Mo-TB, respectively) displayed similar rates of survival at 120 hpf to Larp7-mismatch-injected controls (90%) (Fig. S1C) with mild structural and cardiac abnormalities (see Fig. 4A; Fig. S3). Ventricle diastolic area and the ejection fraction were not different between the two groups (Fig. 4B,C), whereas atrial diastolic area was significantly greater in the hearts of embryos that had been injected with the Larp7 morpholinos compared to controls (Fig. 4D). Larp7-Mo-SB-injected larvae had a significant reduction in *larp7* mRNA by approximately 40% (Fig. 4E) and a reduction in Larp7 protein by 70% (Fig. 4E,F) compared to controls. Larp7-Mo-TB-injected larvae showed an increase in phosphorylation of Ser2 on RNA polymerase II compared to mismatch-treated controls (Fig. 4F), consistent with activation of Cdk9.

### Effects of Cdk9 modulation on cardiomyocyte proliferation

**Fig. 5.**
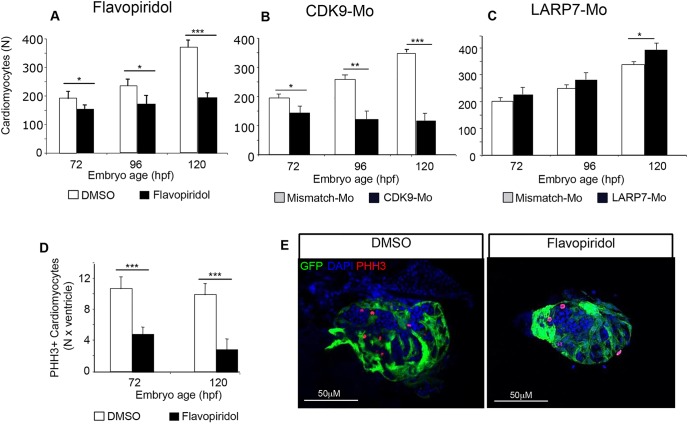
**Effects of Cdk9 modulation on ventricle cardiomyocyte proliferation during early development of the zebrafish heart.** Effects of flavopiridol (A, white bars, controls; black bars, flavopiridol 3 μmol/l) on the total number of cardiomyocytes in the isolated embryonic zebrafish ventricle. Effects of Cdk9 morpholino (B, Cdk9-Mo-SB, Cdk-Mo) and Larp7 morpholino (C, Larp7-Mo-SB, Larp7-Mo) on total cardiomyocyte number in the embryonic ventricle (white bars, controls, appropriate mismatch morpholino; dark bars, splice-blocking morpholino. Effects of flavopiridol on the number of proliferating cardiomyocytes (D, white bars, controls; black bars, flavopiridol 3 μmol/l). Representative confocal images (E) of isolated embryonic *myl7*:GFP hearts stained with DAPI (blue) and for phospho-Histone-H3 (PHH3, red nuclei, marking proliferating cardiomyocytes). Data are mean±s.e.m., *n*=3 experiments, *n*=5 hearts per group, *P<0.05, **P<0.01 ***P<0.001, two-way ANOVA test. Means±s.e.m. are shown in A, B, C and D.

The total number of ventricle cardiomyocytes (VC*t*) increased progressively during development in controls. Larvae that had been exposed to flavopiridol 3 µmol/l (Fig. 5A) showed reduced VC*t* during this same developmental time period. Larvae that had been treated with Cdk9-Mo-SB also showed significantly lower VC*t* compared to mismatch controls with a reduction of 25%, 48% and 70%, respectively, at 72, 96 and 120 hpf (Fig. 5B). We ensured that only dividing cardiomyocytes were included in the counting process by carefully confirming that each phospho-Histone-H3 (PHH3)-positive nucleus colocalised with the DAPI and GFP signals. Exposure to flavopiridol 3 µmol/l significantly reduced the number of mitotic cardiomyocytes (Fig. 5D,E). The ratio of mitotic:total cardiomyocytes at 72 hpf was 2.8% in flavopiridol-treated embryos compared to 5.5% in controls, corresponding to a 55% reduction in the number of PHH3-positive nuclei. In contrast, Larp7-Mo-injected embryos (SB) showed an increased VC*t* at 120 hpf compared to mismatch-injected controls (Fig. 5C).

### Cdk9 modulation modifies GATA-family mRNA abundance in whole larvae

**Fig. 6.**
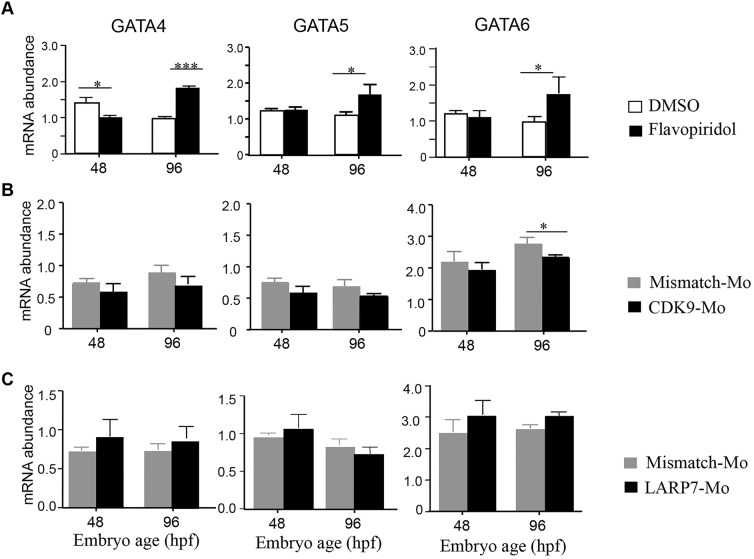
**Pattern of expression of GATA-family genes in whole embryos at 48 and 96 hpf after treatment with flavopiridol or morpholino-mediated knockdown.** For pharmacological inhibition (A), embryos were continuously exposed to vehicle (1% DMSO, white bars) or flavopiridol (3 μmol/l, dark bars) from 24 to 120 hpf. For Cdk9 (B, Cdk9-Mo-SB) or Larp7 (C, Larp7-Mo-SB and -TB) morpholino-mediated knockdown embryos at the one- to two-cell stage were injected with the appropriate mismatch morpholino (white bars) or gene-targeting morpholino (black bars). mRNA was extracted from whole embryos (at least 10 embryos per group) at 48 and 96 hpf. The relative abundance of mRNA for *gata4*, *gata5* and *gata6* was assessed by using qPCR analysis. mRNA levels were normalised to those for β-actin in each case. *n*=3 experiments, *P<0.05, ***P<0.001, two-way ANOVA followed by Bonferroni's post-hoc test. Means±s.e.m. are shown in each figure.

Whole larvae (48 hpf) that had been incubated in flavopiridol showed a reduction in the mRNA levels of *gata4* by 48 hpf with no change in the levels of *gata5* and *gata6* at this developmental stage (Fig. 6A). However, at 96 hpf, larvae that had been treated with flavopiridol showed an increased level of all GATA-family mRNA, with values of 1.5±0.2 for *gata4*, 1.4±0.18 for *gata5* and 1.49±0.3 for *gata6* (mean±s.e.m.).

Larvae that had been treated with Cdk9-Mo-SB did not show a difference in *gata4* and *gata5* at 48 and 96 hpf (Fig. 6B), whereas there was a reduction in *gata6* mRNA abundance at 96 hpf. Larvae that had been treated with Larp7-Mo-TB showed no significant changes in the abundance of mRNA levels for *gata4, gata5* and *gata6* at 48 or 96 hpf (Fig. 6C).

### Cdk9 inhibition impairs recovery from cardiac laser injury

**Fig. 7.**
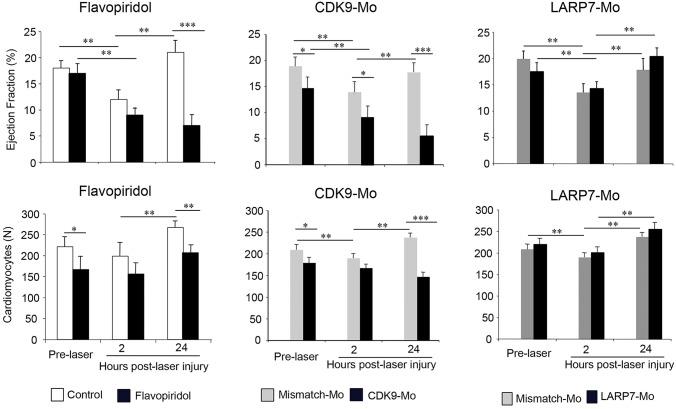
**Effects of Cdk9 modulation on the response of the ventricle to laser injury.** Cdk9 activity was modulated pharmacologically and genetically by incubation in 3 μmol/l flavopiridol and injection of Cdk9- and Larp7-targeting morpholinos (appropriate mismatch controls, grey bars; Cdk9-Mo-SB or Larp7-Mo-SB, dark bars) prior to injury. Laser injury (single pulse to the mid-ventricular cavity) was performed at 72 hpf. Ejection fraction and cardiomyocyte number were assessed pre-laser, and 2 and 24 h post-laser injury (*n*=3 experiments, *n*>10 embryos per experiment, two-way ANOVA test followed by Bonferroni's post-hoc test; *P<0.05, **P<0.01, ***P<0.001). Means±s.e.m. are shown in each figure.

In control larvae, laser injury of the ventricle at 72 hpf resulted in a significant reduction in the ejection fraction and VC*t* at 2 h post laser treatment (Fig. 7). By 24 h post laser treatment, both parameters recovered to baseline levels that were comparable to those before laser injury.

Larvae that had been exposed to flavopiridol or injected with Cdk9-Mo-SB also showed a reduction in the ejection fraction and VC*t* at 2 h post laser treatment; however, both parameters failed to recover to control levels at 24 h post laser treatment (Fig. 7).

Larvae that had been treated with Larp7-Mo-TB showed a significant reduction in the ejection fraction and VC*t* values at 2 h post laser treatment with recovery to control levels at 24 h after laser exposure (Fig. 7).

### Co-injection of Larp7 morpholino and *cdk9* capped RNA attenuates the morphological phenotype in Cdk9-Mo-SB-treated larvae, and restores functional recovery of the ventricle following laser injury

**Fig. 8.**
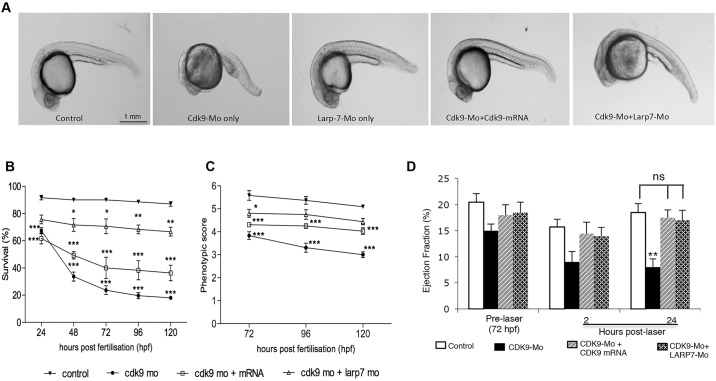
**Rescue of the Cdk9-knockdown phenotype using capped *cdk9* mRNA or Larp7-Mo-SB.** (A–C) Survival and phenotype score in embryos treated with CDK9-Mo-SB (Cdk9-Mo), LARP7-Mo-SB (Larp7-Mo), CDK9-Mo-SB+*cdk*9 mRNA, Cdk9-Mo-SB+Larp7-Mo-SB assessed independently during the first 120 hpf (*n*=3 experiments, *n*>50 embryos per group). (D) Effects of rescuing the Cdk9-knockdown phenotype (*cdk9* mRNA or Larp7-Mo-SB) on recovery of the cardiac function after laser injury (*N*=3, *n*=20–30 embryos per experiment). Two-way ANOVA and Bonferroni post-hoc test; ns, non-significant; *P<0.05, **P<0.01 and ***P<0.001. Means±s.e.m. are shown in B, C and D.

Co-injection of larvae with Cdk9-Mo-SB and Larp7-Mo-SB significantly attenuated the morphological abnormalities observed with Cdk9-Mo-SB alone, with fewer embryos displaying body axis and cardiac abnormalities (Fig. 8A–C). Co-injection of Cdk9-Mo-SB and *cdk9* RNA also significantly rescued the morphological phenotype observed with Cdk9-Mo-SB alone (Fig. 8A–C). There were significantly fewer larvae with small heads, curved tails, string-like hearts and with a lack of tail blood flow. Although the rescue with *cdk9* capped RNA did not completely restore the phenotype to normal, we observed a substantial improvement in the structural and functional cardiac phenotype (Fig. 8C).

Following laser injury, co-injection of Cdk9-Mo-SB with either *cdk9* mRNA (capped) or Larp7-Mo-SB both enhanced recovery from laser injury, with the ejection fraction restored to normal in both co-injected embryo sets within 24 h (Fig. 8D). This compared favourably with the larvae that had been injected with Cdk9-Mo-SB alone, which showed a persistently depressed ejection fraction at 24 h after laser injury.

## DISCUSSION

The zebrafish embryo provides a unique opportunity to assess the role of Cdk9 in cardiomyocyte proliferation by using a highly accurate method of counting cardiomyocytes in whole embryonic ventricles combined with detailed structural and functional assessment of hearts at baseline and after laser-induced injury. We provide evidence that Cdk9 plays a key role in early cardiac development and in cardiomyocyte proliferation. We have shown that Cdk9 action can be pharmacologically inhibited, resulting in dephosphorylation of its target site on the C-terminal domain of RNA polymerase II. Knockdown of *cdk9* transcription by using morpholino technology also resulted in reduced Cdk9 protein expression, leading to reduced phosphorylation of its target site. We have shown that even a modest reduction (about 20%) of Cdk9 protein, insufficient to cause significant structural or developmental abnormalities, has a striking effect on the embryonic heart, resulting in reduced size and function, and a reduced capacity to recover from injury. In contrast, enhancing Cdk9 activity through knockdown of its repressor molecule, Larp7, increased cardiomyocyte proliferation and was associated with normal recovery of the ventricle from laser injury. These findings highlight the importance of Cdk9 in the developing heart and its response to injury. Therefore, Cdk9 represents a fundamental cellular mechanism in the heart that controls transcription and proliferation, and point to its potential importance as a therapeutic target.

Molecular pathways that are linked to CDK9 appear to be more strongly associated with cell growth than control of cell cycle, where other members of the CDK family have greater influence. Previous work has demonstrated that a dominant-negative CDK9 construct blocks ET-1-induced hypertrophy in cultured mouse cardiomyocytes (Sano et al., 2002), confirming a key role for the protein in cardiac hypertrophy.

### CDK9 during embryonic development

In the mouse, cardiac CDK9 activity is highest during embryonic stage (E)16.5, falling by 50% in the neonate and further still to around 15% of that in the embryo by early adulthood (Sano et al., 2002). In the zebrafish heart, quantitative (q)PCR analysis indicates that Cdk9 is most strongly expressed during the first 96 hpf of development, consistent with active cardiomyocyte proliferation. Thereafter, mRNA levels fall only modestly, by around 30%, remaining at around 70% of embryonic expression levels into adult life. This apparent difference could reflect the higher level of cardiomyocyte cell turnover in the zebrafish heart compared with that in the mouse (Soonpaa and Field, 1998).

Although the mechanisms of cell cycle regulation are broadly conserved, the existence and function of Cdk9 in the zebrafish has not been well described. One of the key aspects of our study was to establish that Cdk9 is present and functional in the larval heart, and to characterise its role in normal development. Inhibition of Cdk9 by using flavopiridol produced a significant decrease in Cdk9 activity, as assessed by examining the phosphorylation of its target site Ser2 on RNA polymerase II. Injection of *cdk9*-targeting morpholinos also resulted in reduced Cdk9 expression. These findings confirmed that Cdk9 is involved in the same pathway in the zebrafish as it is in mammalian systems (Nguyen et al., 2001). Treatment with morpholinos targeting *cdk9* produced a number of phenotypic traits that were generally more severe than those observed with flavopiridol treatment, possibly reflecting differences in the time points at which the two forms of treatment were applied during development. Cardiovascular function was affected in both cases, with a reduction in cardiomyocyte proliferation, ventricle ejection fraction and tail blood flow.

### Inhibition of CDK9

CDK9 inhibition is currently being evaluated in pre-clinical and clinical studies for the treatment of a number of different cancers, mainly in combination with other chemotherapeutic agents (Hofmeister et al., 2014; Karp et al., 2010). Although it has been proposed as a treatment for severe cardiac hypertrophy, there are no published trials and no evidence of any ongoing trials of its use in this clinical setting. The proposed mode of therapeutic action of CDK9 inhibitors in cardiac hypertrophy is inhibition of RNA and protein synthesis by limiting gene transcription.

In our study, flavopiridol initially caused a marked reduction in the phosphorylation of its target site on the C-terminal domain of RNA polymerase II (Fig. 2A,C). This effect was relatively transient, despite continuous exposure to the drug, with normalisation of phosphorylation levels at 96 hpf. Simultaneously, there was an unexpected and progressive increase in the levels of Cdk9 protein between 48 and 96 hpf, despite ongoing exposure to the drug (Fig. 2B,C). The lack of specific antibodies for the total levels of zebrafish RNA polymerase II did not allow us to fully exclude the possibility that the levels of RNA polymerase II also changed under our experimental conditions. Following flavopiridol treatment, we observed a small non-significant rise in *cdk9* mRNA abundance at 96 hpf. Taken together, these findings suggest that the initial effect of flavopiridol changed with time, possibly owing to tolerance or desensitisation, or to the use of alternative or redundant Cdk pathways that lead to phosphorylation of the target site. The increase in Cdk9 protein levels is interesting and could be the result of a feedback mechanism that ensures ongoing and adequate phosphorylation of the C-terminal domain of RNA polymerase II to maintain RNA transcription and elongation. Postulating such a mechanism is not unreasonable given the fundamental importance of this pathway to basic cellular function. To our knowledge this is the first evidence of a feedback mechanism linking Cdk9 and its phosphorylation target site on RNA polymerase II.

Flavopiridol also reduced the mRNA abundance of *gata4* in the early stages of development and then later, towards 96 hpf, it produced an increase in the whole GATA family, including *gata4*, *gata5* and *gata6*. This finding is somewhat counter-intuitive because these transcription factors are widely recognised as drivers of cardiomyocyte proliferation, which was also observed to be adversely affected by flavopiridol. *gata4*, in particular, plays a key role in a number of signaling pathways in the heart and is known to be activated following haemodynamic stress (Cappola, 2008). Of specific interest is the fact that *gata4* has been shown to form a complex with CDK9, promoted by the intrinsic histone acetyl-transferase p300 that is also required for the kinase activity of CDK9. This complex is, in turn, required for transcription-pathway activation during cardiomyocyte hypertrophy (Sunagawa et al., 2010). Despite the increasing levels of *gata4*, *gata5* and *gata6* at 96 hpf, cardiomyocyte proliferation did not increase significantly. One possible explanation is that these three transcription factors play a dominant role in cardiomyocyte proliferation in the very early stages of heart development (6–24 h), whereas later in development (24–48 h), other factors are more important in orchestrating the proliferative process.

### Enhancing CDK9 activity

Larp7 specifically binds to and enhances the stability of the complex 7SK-snRNA–HEXIM–CDK9, leading to repression of its action on RNA polymerase II. Knockdown of Larp7 would be expected to destabilise this complex, leading to an increase in CDK9 activity. In contrast to CDK9 inhibition (either with flavopiridiol or direct *cdk9*-targeted morpholino knockdown), Larp7 knockdown provoked an increase in VC*t* that was not associated with an increase in the ventricle size. This suggests a predominantly hyperplastic response. There was no change in the ejection fraction of the ventricle, although we did observe a consistent increase in dimensions of the atrium. The cause for this is not clear, and a contribution resulting from altered ventricular chamber stiffness could have played a role.

### Response to laser injury

Cardiac injury is widely recognised to cause a switch of specific genetic and epigenetic programs (Bergmann and Steller, 2010; Dilworth and Blais, 2011; King and Newmark, 2012; Monaghan et al., 2012; Wang et al., 2012), which also occur in zebrafish (Kizil et al., 2012). The model used here, involving targeted laser treatment of the embryonic ventricle, has similarities to myocardial infarction models in mammals – i.e. it generates a sudden loss of myocardial tissue in a regional distribution. It is highly reproducible and permits the study of both injury and recovery in a very short time period of 24 h. At 2 h after laser injury, there is markedly reduced cardiac function, accompanied by a reduced number of cardiomyocytes and increased apoptosis in the area of injury (Matrone et al., 2013). This functional loss recovers fully within 24 h. Inhibition of Cdk9 activity, with either flavopiridol or morpholinos, resulted in a reduced capacity of the ventricle to recover from injury. This failure to recover was associated with diminished cardiomyocyte proliferation. Although loss of CDK9 action might have influenced a wide range of gene transcription pathways, including apoptosis pathways (Sano et al., 2004), the observed persistent cardiac dysfunction could have been greatly affected by the observed reduction in cardiomyocyte proliferation. Because Cdk9 knockdown caused a persistent reduction in the expression of *gata4*, *gata5* and *gata6*, which are recognised proliferative transcription factors in the heart, we hypothesised that Larp7 knockdown would increase the number of cardiomyocytes. We also hypothesised that this approach would enhance the ability of the ventricle to recover from injury. We showed very clearly that the ventricles of Larp7-Mo-treated embryos had increased numbers of cardiomyocytes at 120 hpf. Subsequently, we showed that we could rescue the CDK9-Mo-SB baseline phenotype with both *cdk9* RNA and also by co-injecting Larp7-Mo. We also showed that co-injection of Larp7-Mo reversed the effects of CDK9-Mo-SB, permitting the embryonic heart to retain its capacity to recover fully from laser injury. As part of a set of validation experiments, we co-injected CDK9-targeting morpholino with the mismatch Larp7 morpholino. This co-injection did not rescue the embryo phenotype. Only the co-injection of the Cdk9- and Larp7-targeting morpholinos rescued the Cdk9-targeting morpholino embryonic phenotype. In addition, we prepared the solution immediately before the injection, to reduce as much as possible the formation of non-specific complexes. However, we cannot completely exclude the possibility of non-specific complex formation interfering with the action of the Cdk9-Mo-SB. Larp7 knockdown alone did not significantly affect cardiac recovery from laser injury.

The extent of Cdk9 protein knockdown resulting from morpholino treatment was around 20%, indicating the persistent availability of Cdk9 for modulation following Larp7-Mo treatment. In addition, the dynamic sharing of Cdk9 between the two complexes in which Cdk9 is involved – i.e. the Cdk9–Cyclin active fraction and Cdk9–Larp7–Hexim–7SK inactive fraction, represents a functional balance that can be perturbed. Indeed, we believe that following Larp7 knockdown, more Cdk9 enzyme is released from the inactive complex leading to increased activity. In mammals, the inactive P-TEFb complex includes HEXIM and 7SK, and co-inhibiting two or more of these CDK9 repressors could be a possible short-term therapeutic approach to enhance recovery from injury in the heart.

However, forcing mammalian cardiomyocytes to re-enter the cell cycle could activate mechanisms controlling inappropriate cell cycle entry in otherwise quiescent cells. For instance, a foreseeable obstacle to this approach in cardiomyocytes is that E2F-1, which provokes G1 exit and DNA synthesis (Kirshenbaum et al., 1996), could also induce cardiomyocyte p53-dependent (Kowalik et al., 1995) or -independent (Agah et al., 1997) apoptosis.

In this case, therapeutic interventions should include ad-hoc modulation of anti-apoptotic genes, such as *BCL-2* (Kirshenbaum and de Moissac, 1997).

Clearly, further understanding of the factors and pathways controlling CDK9 activity is required, and these pathways clearly merit further study as possible therapeutic targets to address diseases associated with acute and chronic heart injury.

### Future perspectives

Our findings, although limited to the zebrafish embryonic heart, have refocused on the importance of CDK9 as a potential therapeutic target for human disease. There is clearly a need for further studies in mammalian cardiac model systems. A first step would be to explore CDK9 expression patterns in neonatal versus adult hearts and to analyse its role in regenerative capacity (Porrello et al., 2011). It would also be very important to understand whether a heart-specific CDK9 isoform exists, which would permit development of drugs targeted at the action of CDK9 in the heart, thus avoiding off-target effects. De-repression of CDK9 through LARP7 knockdown or inhibition is an intriguing approach, and one which could promote cell survival and enhance proliferation. Although this could potentially lead to carcinogenesis, short-term use during periods of acute injury, such as after myocardial infarction, merit further study.

In conclusion, we have shown that the activity of CDK9 and its repressor LARP7 can be modified to impact on cardiac growth and development, specifically impacting on cardiomyocyte proliferation. Short-term de-repression of CDK9 represents an unexplored mechanism with the potential to promote functional recovery of the heart following injury through a mechanism that is associated with enhanced cardiomyocyte proliferation.

## MATERIALS AND METHODS

### Ethical approval

All experiments were approved by the local ethics committee and conducted in accordance with the United Kingdom Animals (Scientific Procedures) Act 1986 in an approved establishment.

### Zebrafish maintenance

Zebrafish husbandry, embryo collection and maintenance were performed according to accepted standard operating procedures (Nüsslein-Volhard and Dahm, 2002). The cardiac myosin light chain 2:GFP transgenic line [Tg(cmlc2:GFP)] (Burns et al., 2005) was used for all experiments; larvae were maintained at 28.5°C on a 14-h-light–10-h-dark cycle and staged according to Kimmel et al (1995). Larvae were maintained in egg water until dechorionated, and then in embryo medium (Westerfield, 2000). Embryos were anaesthetised in a solution of Tricaine 20 µmol/l (ethyl 3-aminobenzoate methanesulfonate, Sigma-Aldrich) or euthanised with an overdose of the same compound. All experimental procedures were performed at room temperature (23°C).

### Morpholino injections

Suppression of Cdk9 and Larp7 expression was achieved using antisense morpholinos (Gene Tools) designed against the splice donor between exon 3 and intron 3, or targeting the mRNA AUG translational start site.

Morpholino sequences were as follows: Cdk9-Mo-SB (accession number NM_212591.1) 5′-CTTTCTTCCCCATTCTTTTACGTGG-3′; Cdk9-Mo-TB 5′-CCTACGTCGCGCTGTTTTGGCCTTC-3′; Cdk9 control mismatch 5′-CTTTgTTgCCgATTgTTTTACcTGG-3′; Larp7-Mo-SB (accession number NM_199930.1) 5′-TCATCTCCATACTAAACCAAACTGT-3′; LARP7-Mo-TB 5′-TACTTTCACACAGTTGCGTTCTGCT-3′); Larp7 control mismatch 5′-TgATgTCCATAgTAAACgAAACTcT-3′. All mismatch morpholinos represent protein-specific oligonucleotides where mentioned in the text and figure legends.

0.5 nl of morpholino solution (100 µmol/l for Cdk9 and 200 µmol/l for Larp7) was injected in one- to two-cell-stage larvae, just beneath the blastoderm using a pulled glass pipette using a standard injector (IM300 Microinjector, Narishige, Japan). Successful injection was assessed under a fluorescence microscope by using the red tag lissamine at the 3′ end of the morpholino oligonucleotides.

### Rescue of Cdk9 knockdown by *cdk9* mRNA

To determine whether the effect of the Cdk9-targeting morpholino was specifically due to loss of Cdk9, we performed a rescue experiment by co-injecting CDK9-Mo with capped *cdk9* RNA. This capped RNA was produced using an IMAGE clone encoding *Danio rerio cdk9* cDNA (Source Bioscience), which was sub-cloned into pNR-LIB (Source Bioscience). Following linearisation with *Xho*I restriction enzyme (Applied Biosystems), capped *cdk9* RNA was transcribed using an mMessage mMachine Kit (Ambion) according to the manufacturer's instruction. A bolus of 1 nl of solution containing CDK9-targeting morpholino at 100 µmol/l and 1.25 ng of *cdk9* RNA was injected into each egg.

### Rescue of the effects of Cdk9 knockdown through Larp7 knockdown

Co-injection of Cdk9-Mo-SB and LARP7-Mo was performed to assess whether the phenotype and the response to laser injury observed in Cdk9-Mo-SB-treated larvae could be rescued by Larp7 knockdown. A bolus of 1 nl of solution containing Cdk9-targeting morpholino at 100 µmol/l and Larp7-targeting morpholino 200 µmol/l was injected in each egg.

### Pharmacological treatment of larvae

Flavopiridol is recognised as a potent ATP competitive kinase inhibitor for CDK9 (Bosken et al., 2014; Baumli et al., 2008), that is now in clinical trials (Wang and Fischer, 2008). Zebrafish larvae (24 hpf) were placed in embryo medium containing flavopiridol 3 µmol/l (Sigma-Aldrich) diluted in 1% DMSO. Solutions were replaced at 48, 72 and 96 hpf. Control embryos were exposed to 1% DMSO. The drug concentration of 3 µmol/l was selected after a series of experiments assessing the concentration of flavopiridol because it resulted in minimum toxicity to the whole embryo while also resulting in a significant decrease in Cdk9 activity, confirmed by reduced phosphorylation of the residue Ser2 on RNA polymerase II.

### Assessment of ventricular function

Videos of the beating heart were captured by using a video camera (IonOptix CCD100 MyoCam™, Dublin, Ireland), mounted on a microscope (Axioscope II MOT Plus, Zeiss) linked to a computer. Ventricle end-systolic and end-diastolic area were measured using image analysis software (ImageJ). Fractional-area change (ejection fraction) was estimated by subtracting ventricular systolic area from diastolic area, expressed as a percentage of diastolic area, as previously described (Shu et al., 2003).

### Histology and ventricle cardiomyocyte number

VC*t* and proliferating ventricular cardiomyocyte number, counted as DAPI- and PHH3-positive nuclei, respectively, were assessed in whole-mount isolated embryonic hearts, as previously described (Matrone et al., 2015). Larvae were euthanised in 1 mM tricaine and fixed in 4% paraformaldehyde (PFA, Sigma-Aldrich). Microdissected hearts were pre-incubated in proteinase K (10 µg/ml) to allow permeabilisation, washed in PBS and Triton-X100 (0.1%) and then blocked in bovine serum albumin 5% in PBS for 3 h before being incubated with an anti-PHH3 antibody (Millipore 05-670; rabbit, 1:200), followed by incubation with anti-rabbit IgG antibody (Alexa Fluor, Dako, 1:500). Subsequently, hearts were incubated in DAPI (Sigma-Aldrich, 1:1000) for 1 h, washed in PBS and then mounted in glycerol 100%. Confocal microscopy (Leica SP5) was used to capture *z*-stack images of isolated heart ventricles at 3 µm intervals. The VC*t* and the number of mitotic ventricular cardiomyocytes were counted using ImageJ software, by marking each nucleus with a tag while moving progressively through the *z*-stack. Only cardiomyocytes were included in the counting process by ensuring that each nucleus was located within a GFP-positive region of the heart. The atrium and bulbous arteriosus were excluded from counting. Counting was performed by a single individual, blinded to the original treatment of the heart. Intra-observer variation for a sample of 25 hearts was ±4.5%.

Haematoxylin and eosin (H&E) staining of whole larvae was performed. Serial 4 μm sagittal sections were stained according to standard protocols (Sabaliauskas et al., 2006).

### Heart laser injury

Laser injury of the zebrafish embryonic heart was induced using a XYclone laser (Hamilton-Thorne, USA), using a technique that has been previously reported by our group (Matrone et al., 2013). The protocol for laser injury and recovery is summarised in Fig. S4. Briefly, larvae at 72 hpf were anaesthetised using tricaine at 20 µmol/l (Sigma-Aldrich) and placed on a plain glass microscope slide in a minimal amount of water. Under standard white light microscopy (Axioscope II MOT Plus, Zeiss), a laser pulse was delivered to the midpoint of the ventricle (see Movie 1). The average energy delivered per pulse was 0.9 mJ over a duration of 3 ms (300 mW), directed at an area approximately 10 μm in diameter. After injury, each embryo was immediately placed into embryo medium to recover for a further 48 h. Control larvae, kept in identical conditions and manipulated in the same way, received laser injury to the distal tail-fin.

### Gene expression

qPCR was used to assess the mRNA levels of *cdk9*, *larp7*, *gata4*, *gata5* and *gata6*, following morpholino injection or flavopiridol exposure.

RNA extraction and reverse transcription were performed by using Qiagen RNeasy mini kit (Qiagen).

Larvae were placed in a 1.5-ml Eppendorf tube, euthanised with tricaine overdose and stored in RNAlater (Life Technologies) or, for immediate RNA extraction, placed directly in 600 µl of buffer RLT. Two small metallic beads that had been previously autoclaved and wiped with RNase Zap (Life Technologies) were added to each Eppendorf. Efficient disruption and homogenisation was performed by milling for 45 s at 30 Hz (Mixer Mill 301 model, Retsch, Haan, Germany) while keeping the larvae homogenate cold at all times. Beads were removed, and the samples were centrifuged at 13,000 ***g*** for 3 min at 4°C. The supernatant was placed in a new 2-ml Eppendorf tube, and the pellet was discarded. Supernatant was added with an equal volume of 70% ethanol, mixed and placed in the RNeasy spin column subjected to centrifugation (12,000 ***g***, 30 s, 4°C). The eluate was discarded.

The DNase treatment step was performed subsequently to eliminate genomic DNA contamination. After wash with buffer RW1 (350 μl) and centrifugation for 15 s, 10 μl DNase I stock solution, previously diluted in 70 μl of buffer RDD, were added to the spin column and incubated for 15 min at room temperature. The column was then washed with buffer RW1 (700 μl) and buffer RPE (500 μl). In each case, the eluate was discarded following centrifugation (12,000 ***g***, 15 s, 4°C). Buffer RPE (500 μl) was added to wash the membrane, followed by a further centrifugation step (12,000 ***g***, 2 min, 4°C). The spin column was then placed in a fresh 2 ml collecting tube and subjected to centrifugation (16,000 ***g***, 1 min, 4°C) to eliminate any buffer RPE carryover. The spin column was then placed in a fresh 1.5-ml Eppendorf, RNase-free water (30 μl) was added, and the RNA was eluted by using centrifugation (12,000 ***g***, 1 min, 4°C). Eluted RNA was stored at −80°C.

RNA was quantified using a Nanodrop Spectrophotometer (Thermo Fisher) in 1 μl of RNA sample. Concentration was determined by the absorbance at 260 nm wavelength (A_260_), and the purity assessed by the ratio of RNA/DNA (A_260_/A_280_), which was deemed acceptable if between 1.9 and 2.1, and a 260/230 ratio (some contaminants, e.g. phenol, absorb at 230 nm) >2.2.

Quality of RNA was assessed by electrophoresis on agarose (Lonza) gel (1% w/v in 0.5× TBE). Samples (2 μl) were prepared by adding loading dye (Promega) 1 in 5 diluted in distilled water. Samples were incubated at 85°C for 3 min to denature RNA before running on the gel (100 V, 1 h) with a size marker (New England Biolabs). RNA integrity was assessed on the basis of 18S and 28S ribosomal RNA (rRNA) bands. RNA integrity was deemed satisfactory if clear 28S and 18S rRNA bands were present without smearing, and if the 28S rRNA band was approximately twice as intense as the 18S rRNA band.

RNA was reverse transcribed in cDNA by using high capacity cDNA reverse transcription kit (Applied Biosystems).

For each sample, a volume containing 2 µg of RNA was pipetted into a 0.2 ml microfuge, and made up to 10 μl with nuclease free water, then 10 μl of RT master mix (2×) was added. Two negative controls were prepared as above, one with water instead of RNA to identify any RNA contamination in the reagents, the second without the reverse transcriptase enzyme in order to detect the presence of contamination by genomic DNA. Samples were incubated (25°C, 10 min; 37°C, 120 min; 85°C, 5 min; 4°C, ∞) in a PCR machine, before being chilled to 4°C. The resultant cDNA was stored at −20°C.

To enable quantitation of gene expression, two housekeeping genes were chosen based on their stability in respective experiments. Ef1α was used during normal embryonic development, whereas β-actin was used during embryonic exposure to flavopiridol or treatment with morpholinos.

Primers were designed to match probes within the Roche Universal Probe Library (https://www.roche-applied-science.com). cDNA samples were heated for initial denaturation (95°C, 5 min), then underwent 50 cycles of PCR amplification, which comprised denaturation (95°C, 10 s), annealing (60°C, 30 s) and elongation (72°C, 1 s). Upon completion of the PCR program, samples were cooled (40°C, 30 s). For all the samples, amplification curves were plotted (*y*-axis fluorescence, *x*-axis cycle number). Triplicates were deemed acceptable if the standard deviation of crossing point (Cp) was <0.5 cycles. Relative quantification was calculated using the ΔCT method and Light Cycler software.

### Protein analysis

Western blotting followed by densitometric quantification of the membrane bands (ImageJ software) was used for semi-quantitative analysis of proteins. Anti-Cdk9 antibody was produced in rabbit (C12F7, Cell Signaling Technology; 1:200 in PBS); an antibody recognising RNA polymerase II phosphorylated at Ser2 was produced in mice (ab24758, Abcam; 1:500 in PBS); Larp7 was recognised using a synthetic peptide (AV40848, Sigma-Aldrich; 1:1000 in PBS). To correct for variations in the amount of total protein loaded onto the gel, the levels of target proteins were normalised to those of protein β-tubulin (ab6046, Abcam).

Embryo chorion and yolk sac were surgically removed using fine forceps (Dumont #5). Dechorionated and de-yolked larvae were transferred into fresh, cooled PBS solution and rinsed twice, and transferred into a 1.5-ml Eppendorf tube. Samples were microfuged for 1–2 min, and supernatant was removed. About 100–150 μl RIPA lysis buffer (Millipore), that had been previously diluted in deionised water from a 10× stock, was added and homogenised with a microfuge pestle for 30 s, and then sonicated for 30 s at 5–8 μW, until the embryo sample was no longer visible. Samples were kept in ice for 30 min, then were briefly vortexed and microfuged for 1–2 min. Supernatant was collected and then placed in a new 1.5 ml tube, and the pellet was discarded.

The protein concentrations of zebrafish larvae homogenates were determined colorimetrically using a Bio-Rad protein assay kit (Bio-Rad) and the Bradford method of detection.

An appropriate amount of protein sample containing 20 μg of total protein was taken into a fresh Eppendorf tube, and 4 μl of 4× Laemmli buffer and deionised water were added to make a final volume of 16 μl. Samples were mixed and heated at 95°C for 5 min.

Samples were separated by electrophoresis on a NuPAGE^®^ Novex^®^ 4–12% Bis-Tris gels, 1.0 mm thick (Life Technologies). In addition to the samples, 5 μl of Novex^®^ Sharp Pre-stained Protein Standard (Life Technologies) was also loaded to identify the molecular mass of bands. Gels were run at a voltage 100 V for 30 min and then at 150 V for another 60 min.

Gels were transferred onto nitrocellulose membranes Protran (Sigma-Aldrich) using the Electrophoretic Transfer Cell (Bio-Rad Laboratories). A gel sandwich was prepared within the cassette, comprising fibre pad, blotting paper (both from Bio-Rad Laboratories), gel, nitrocellulose membrane, filter paper and fibre pad. The gel sandwich cassette was placed within the transfer module and tank, which was then filled with Towbin transfer buffer (Tris-HCl 0.025 M, glycine 0.192 M, pH 8.6±0.1). The transfer was run at 100 V and 300 mA for about 3 h at 4°C. Nitrocellulose was then stained in Red Ponceau (Sigma-Aldrich) for 5 min and then washed in water to check for the presence of protein, and the remaining gel was stained in Coomassie Blue R-250 0.5% (Bio-Rad Laboratories) for 1 h, followed by several washes in decolouration solution to assess that good transfer had occurred.

After transfer, nitrocellulose membrane was blocked with 20 ml of 5% dried skimmed milk (Marvel Premier Brands, Lincolnshire, UK) in TBS Tween [20 mM Tris-Cl (0.2 M Tris-Cl, pH 7.6), 137 mM sodium chloride, 0.1% Tween 20 (Polyoxyethylenesorbitan monolaurate, BDH, Poole, UK)] on a platform shaker for 1 h at room temperature. Then the membrane was washed three times (5 min each) with PBS-Tween 0.1% and then incubated in primary antibody at a concentration according to antibody manufacturer (usually 1:500–1:1000) in 3% BSA (Sigma-Aldrich) overnight at 4°C. The membrane was washed three times in PBS-Tween 0.1% prior to incubation with a secondary antibody, linked to horseradish peroxidase at a concentration of 1:10,000 in 5% dried skimmed milk for 1 h. A final series of washes was performed as above, before the blot was developed.

Nitrocellulose membrane was exposed for 1 min to the enhanced chemiluminescence solution (ECL; Amersham) following the manufacturer's instructions. Excess ECL substrate was removed from the membrane by touching the edge of the membrane to a piece of tissue paper. Then the membrane was placed down on a film layer, which had been arranged inside a film cassette. The bubbles were carefully removed from the membrane, which was then covered with another film layer, fixed onto the cassette with tape and exposed to photographic film (BioMax XAR Film Kodak, Sigma-Aldrich) for an adequate exposure time. The film was developed and immunoreactivity (band density) was quantified by using densitometry (online source: http://rsbweb.nih.gov/ij/docs/user-guide.pdf) using ImageJ.

### Defining the whole embryo and cardiac phenotype

Whole embryo and cardiac phenotype following treatments and following rescue were described on the basis of morphologic and functional characteristics under bright-field microscopy. The embryo phenotype was assessed using a simple six point scoring system – one point was allocated for each of the following features: normal heart rate, normal head shape and size, normal body axis, normal tail blood flow, absence of pericardial oedema and absence of blood pooling. If these were all normal in any single embryo, then this animal was scored as 6. Further detailed phenotype characterisation was undertaken independently within our laboratory for each of the three Cdk9 manipulations. In addition to the above characteristics, this assessment also included body angle as a measure of developmental stage, where 0–5% was considered normal, curved was 5–50% and marked curvature was >50%. At least four different clutches of larvae were assessed under each of the treatment groups.

### Statistical analysis

Experiments were performed in triplicate with, on average, 20–30 larvae per experiment, unless otherwise stated. Data are presented as mean±s.e.m. Statistical analyses were performed using GraphPad Prism 5. One-way or two-way repeated measures ANOVA followed by Bonferroni post-hoc test were used to compare means within and between groups. *P*-values <0.05 were considered significant.
